# Body size and its implications upon resource utilization during human space exploration missions

**DOI:** 10.1038/s41598-020-70054-6

**Published:** 2020-08-14

**Authors:** Jonathan P. R. Scott, David A. Green, Guillaume Weerts, Samuel N. Cheuvront

**Affiliations:** 1 KBR, 511147 Cologne, Germany; 2 Space Medicine Team, European Astronaut Centre, European Space Agency, 51147 Cologne, Germany; 3 Centre of Human and Applied Physiological Sciences, King’s College London, London, SE1 1UL UK; 4 Sports Science Synergy, LLC, Franklin, MA 02038 USA

**Keywords:** Metabolism, Physiology, Respiration

## Abstract

The purpose of this theoretical study was to estimate the effects of body size and countermeasure (CM) exercise in an all-male crew composed of individuals drawn from a height range representative of current space agency requirements upon total energy expenditure (TEE), oxygen (O_2_) consumption, carbon dioxide (CO_2_) and metabolic heat (H_prod_) production, and water requirements for hydration, during space exploration missions. Using a height range of 1.50- to 1.90-m, and assuming geometric similarity across this range, estimates were derived for a four-person male crew (age: 40-years; BMI: 26.5-kg/m^2^; resting VO_2_ and VO_2max_: 3.3- and 43.4-mL/kg/min) on 30- to 1,080-d missions, without and with, ISS-like CM exercise (modelled as 2 × 30-min aerobic exercise at 75% VO_2max_, 6-d/week). Where spaceflight-specific data/equations were not available, terrestrial data/equations were used. Body size alone increased 24-h TEE (+ 44%), O_2_ consumption (+ 60%), CO_2_ (+ 60%) and H_prod_ (+ 60%) production, and water requirements (+ 19%). With CM exercise, the increases were + 29 to 32%, + 31%, + 35%, + 42% and + 23 to 33% respectively, across the height range. Compared with a ‘small-sized’ (1.50-m) crew without CM exercise, a ‘large-sized’ (1.90-m) crew exercising would require an additional 996-MJ of energy, 52.5 × 10^3^-L of O_2_ and 183.6-L of water, and produce an additional 44.0 × 10^3^-L of CO_2_ and 874-MJ of heat each month. This study provides the first insight into the potential implications of body size and the use of ISS-like CM exercise upon the provision of life-support during exploration missions. Whilst closed-loop life-support (O_2_, water and CO_2_) systems may be possible, strategies to minimize and meet crew metabolic energy needs, estimated in this study to increase by 996-MJ per month with body size and CM exercise, are required.

## Introduction

To sustain humans in space requires the construction of a protective habitat and the generation (and maintenance) of environmental conditions consistent with life. Any habitat, be it a transit vehicle, orbital outpost such as the International Space Station (ISS) in Low Earth Orbit, or future surface habitat, must not only protect crewmembers from the near vacuum of space, but also the extremes of temperature and other space-specific risks including radiation and micrometeorites. Furthermore, appropriate ‘life-support’ must be provided (i.e. oxygen [O_2_], water and food), in addition to the management/removal of the by-products of human metabolism (carbon dioxide [CO_2_], water vapour, metabolic heat, urine, and faeces). On ISS, the provision of life-support is achieved through a combination of supply (and regular re-supply) from the ground (e.g. the Russian ‘Progress’ expendable cargo and SpaceX’s ‘Dragon’ supply vehicles), and a range of on-board technologies that both manage the internal atmosphere and, increasingly, re-use or recycle by-products (e.g. splitting of CO_2_ to generate O_2_ and partially [70%] efficient recycling of urine for potable water)^1,2^.

However, future space exploration will once again require humans to venture beyond Low Earth Orbit^3^, rendering re-supply significantly more difficult, especially in the case of deep space (i.e. beyond the Lunar orbit) exploration missions. Moreover, in the short- to medium-term, exploration transit vehicles and lunar orbital (and possibly surface) habitats will be markedly smaller than ISS, which currently has a habitable volume of 338-m^3^ ^4^ and typically sustains six, and transiently as many as 15, crew members. In contrast, the National Aeronautics and Space Administration (NASA)’s ‘Orion’ Multi-purpose Crew Vehicle is designed to sustain a crew of four for up to 21-days in a habitable volume of only 8.95-m^3^ ^5^. Furthermore, the current concept of the Lunar Gateway or ‘Gateway’, formerly known as the Deep Space Gateway, a space station where crew may spend up to 30-d in a Cis-Lunar orbit, envisions just two habitation modules^6^. As such, the influence of human metabolism (i.e. depletion of O_2_, and accumulation of CO_2_, heat and water vapour) on the internal atmosphere will potentially be more acute (i.e. depletion/accumulation will be more rapid within a smaller volume of air).

When selecting astronauts for missions to ISS, body ‘size’ is not currently a significant operational consideration. The only anthropometric requirement is that applicants’ stature falls within a specified range (1.495- to 1.905-m; 58.9″ to 74.8″)^7–9^, but this is purely for compatibility with existing hardware, not least the "Kazbek" seat pan liner of the Soyuz transit vehicle. As a result of the relative ease of supply/re-supply and large habitable volume of ISS, the effect of body size on provision of life-support, including the removal of metabolic by-products for the six-person crew is small. That said, even with a nominal ISS crew of six, CO_2_ levels generally range between 0.3 and 0.7% CO_2_ (2.3- to 5.3-mmHg) with a mean of 0.5% CO_2_, equivalent to 16 times that in ambient air at sea level. Furthermore, CO_2_ levels are location-specific depending on the presence of crew members and the relative efficacy of ventilation fans required to reduce expired air bubbling due to the absence of natural convection, with hourly means of up to 0.7% CO_2_ reported^10^. This increased ambient concentration of CO_2_ (and the resulting hypercapnia) is proposed to be a contributor to the high prevalence of headache^11^ and the spaceflight-associated neuro-ocular syndrome observed in ISS astronauts^12^. Interestingly, recent evidence suggests that pre-flight body weight and anthropometrics (chest and waist circumference) may predict microgravity-induced ocular changes associated with this syndrome^13^.

In contrast, it is well established that human (basal) metabolism is, in absolute terms, proportional with body size, reflected in larger individuals possessing higher resting O_2_ consumption (VO_2_), as well as CO_2_ (VCO_2_) and metabolic heat production^14^. Likewise, assuming equal aerobic fitness (maximal oxygen uptake [VO_2max_] relative to body mass), larger individuals will require a greater amount of energy, and thus consume more O_2_, and produce more CO_2_ and metabolic heat, than smaller people at the same relative exercise intensity (e.g. 75% VO_2max_)^15^. Although minute-to-minute these differences may be relatively small, when accumulated over the course of a space mission, they could become substantial, particularly if, as is the case on ISS, regular exercise is performed^16^. In fact, whilst life-support resource constraints are features of other closed (i.e. artificially sustained) environments, such as submarines and polar bases, they are not associated with the high levels of countermeasure (CM) exercise performance in space necessitated to mitigate (to some degree) the deleterious effects of microgravity^16^. Thus, future human space exploration missions may well present the ‘perfect storm’, combining high levels of exercise performance (and thus metabolic cost) in conditions of extreme life-support constraint.

As such, the purpose of this theoretical study was to estimate the effect of body size and CM exercise in a crew of four^5^ composed of individuals drawn from a height range representative of space agency requirements^7–9^ upon total energy expenditure (TEE), O_2_ consumption, CO_2_ and metabolic heat production, and water requirements for hydration, during exploration missions of increasing duration. With physiological differences between males and females in terms of body composition and metabolism^17^, and responses to the spaceflight environment^18^, this initial study focused only on males. In addition, as the operational approach to the use of in-flight CM exercise during exploration missions is yet to be confirmed, to explore the effects of body size and CM exercise, this paper considered two hypothetical scenarios:

1. Male crew living in microgravity, but performing no in-flight CM exercise;
2. Male crew living in microgravity and performing CM exercise comparable in volume to that currently employed on ISS^16^.

## Results

### Characteristics of theoretical astronaut population

Based on the study assumptions and calculations used, the characteristics of the theoretical male astronaut populations are shown in Table 1.

**Table 1:** Characteristics of the theoretical astronaut populations.

Stature (m)	1.50	1.60	1.70	1.80	1.90
BM (kg)	59.6	67.8	76.6	85.9	95.7
BSA (m^2^)	1.54	1.71	1.88	2.06	2.24
VO_2max_ (L/min)	2.59	2.94	3.32	3.73	4.15
Rest					
RMR (MJ/d)	5.78	6.44	7.13	7.85	8.60
NEAT (MJ/d)	2.31	2.58	2.85	3.14	3.44
VO_2_ (L/min)	0.197	0.224	0.253	0.283	0.316
VCO_2_ (L/min)	0.155	0.176	0.199	0.223	0.249
Basal M_prod_ (J/s)	65.7	74.8	84.4	94.7	105.5
Basal fluid needs (L/d)	2.63	2.74	2.86	2.99	3.13
CM exercise @ 75% VO_2max_					
VO_2_ (L/min)	1.94	2.21	2.49	2.79	3.11
VCO_2_ (L/min)	1.74	1.98	2.24	2.51	2.80
EE (kcal/min)	9.6	10.9	12.3	13.8	15.4
M_prod_ (J/s)	667	759	857	960	1,070
SR (mL/min)	10.1	11.7	13.4	15.2	17.1

*BM* body mass; *BSA* body surface area; *VO*_*2max*_ maximal rate of oxygen uptake, *RMR* resting metabolic rate, *NEAT* non-exercise activity thermogenesis, *VO*_*2*_ rate of oxygen consumption, *VCO*_*2*_ rate of carbon dioxide production, *CM* countermeasure, *EE* energy expenditure, *M*_*prod*_ rate of metabolic heat production, *SR* sweat rate. See main text for definition of assumptions.

Total energy expenditure, O_2_ consumed, CO_2_ and metabolic heat produced, and fluid lost through sweating, during a *single* bout (30-min at 75% VO_2max_) of CM exercise are shown in Table 2.

**Table 2:** Estimated total energy expenditure (EE), oxygen (O_2_) consumed, and carbon dioxide (CO_2_), metabolic heat (H_prod_) and sweat produced, during a *single* bout (30-min at 75% VO_2max_) of countermeasure exercise by a theoretical male crew member.

Stature (m)	1.50	1.60	1.70	1.80	1.90
EE (MJ)	1.28	1.45	1.64	1.84	2.05
O_2_ (L)	61.7	70.2	79.3	88.9	99.0
CO_2_ (L)	55.4	63.1	71.2	79.8	88.9
H_prod_ (kJ)	1,200	1,366	1542	1729	1926
Sweat (mL)	303	350	401	455	513

See main text for definition of assumptions.

24-h values for TEE, O_2_ consumed, and CO_2_, metabolic heat and sweat produced by the theoretical populations, without and with CM exercise, are shown in Table 3. Body size alone increased 24-h TEE (+ 44%), O_2_ consumption (+ 60%), CO_2_ production (+ 60%), heat production (+ 60%) and water requirements (+ 19%). With CM exercise, the increases were + 29–32%, + 31%, + 25%, + 42% and + 23–33% across the body size range.

**Table 3:** 24-h values for theoretical male astronaut populations without, and with, the use of ISS-like countermeasure (CM) exercise (modelled as two bouts of 30-min of cycle ergometry at 75% VO_2max_).

Stature (m)	1.50	1.60	1.70	1.80	1.90
No exercise					
TEE (MJ)	8.9	9.9	10.8	11.9	12.9
O_2_ (L)	397	451	510	571	636
CO_2_ (L)	313	356	401	450	501
H_prod_ (MJ)	5.7	6.5	7.3	8.2	9.1
Water requirements (L)	2.63	2.74	2.86	2.99	3.13
CM exercise					
TEE (MJ)	11.9 (+ 29)	12.8 (+ 29)	14.1 (+ 30)	15.5 (+ 31)	17.0 (+ 32)
VO_2_ (L)	494 (+ 31)	562 (+ 31)	634 (+ 31)	711 (+ 31)	792 (+ 31)
VCO_2_ (L)	423 (+ 35)	482 (+ 35)	544 (+ 35)	610 (+ 35)	679 (+ 35)
H_prod_ (kJ)	8.1 (+ 42)	9.2 (+ 42)	10.4 (+ 42)	11.6 (+ 42)	13.0 (+ 42)
Water requirements (L)	3.23 (+ 23)	3.44 (+ 26)	3.67 (+ 28)	3.90 (+ 30)	4.16 (+ 33)

*TEE*, total energy expenditure, *O*_*2*_ total oxygen consumed, *CO*_*2*_ total carbon dioxide produced, *H*_*prod*_ total metabolic heat produced. Numbers in brackets indicate the difference (%) between data without, and with CM exercise. See main text for definition of assumptions.

### Energy expenditure

**Figure 1:**
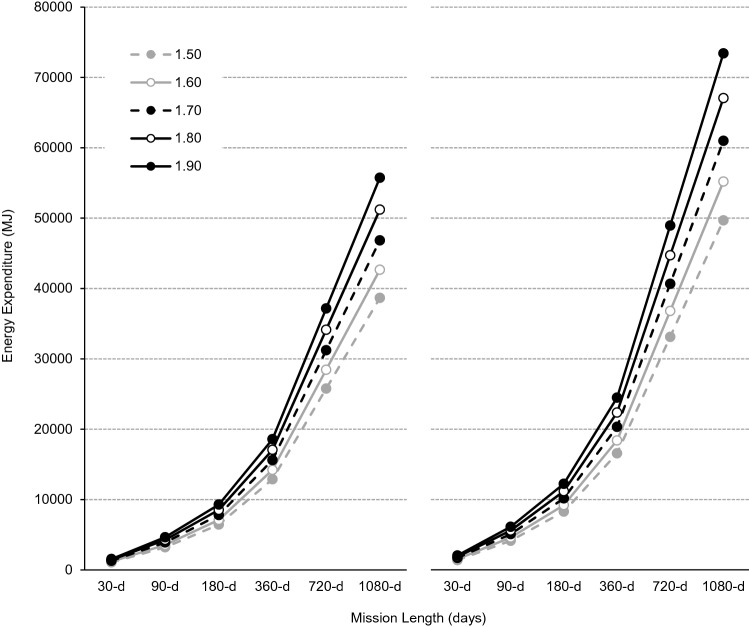
Total energy expenditure (MJ) without (left panel) and with (right panel) countermeasure exercise during exploration missions of 30-, 90-, 180-, 360-, 720- and 1,080-d for a four-person crew based on theoretical male astronaut populations with statures of 1.50-m (broken grey line, filled circles), 1.60-m (solid grey line, open circles), 1.70-m (broken black line, filled circles), 1.80-m (solid black line, open circles) and 1.90-m (solid black line, filled circles).

For a four-person all-male crew without, and with, the performance of CM exercise, body size increased TEE from 1,074-MJ for a ‘small-sized’ (1.50-m) crew to 1548-MJ for a ‘large-sized’ (1.90-m) crew during a 30-d mission, and from 38,659-MJ to 55,742-MJ during a 1,080-d mission (Fig. 1). For a 30-d mission, CM exercise alone increased TEE by 306-MJ for a small-sized crew and to 491-MJ for a large-sized crew, and by 11,019-MJ and 17,680-MJ for a 1,080-d mission. Compared with a small-sized crew performing no CM exercise, a large-sized crew performing ISS-like CM exercise require an additional 966-MJ per month (Table 4).

**Table 4:** Absolute increase in energy expended, oxygen (O_2_) consumed, carbon dioxide (CO_2_) and heat produced (H_prod_), and water required for hydration, during 30-d and 1,080-d missions resulting from the increase in body size *alone* between a ‘small-sized’ (1.50-m) and ‘large-sized’ (1.90-m) male crew, and the use of countermeasure (CM) exercise.

	Increased (1.50- to 1.90-m) body size (without CM exercise)		Use of CM exercise	
	30-d	1,080-d	30-d (1.50–1.90-m)	1,080-d (1.50–1.90-m)
Energy (MJ)	+ 475	+ 17,083	+ 306–491	+ 11,019–17,680
O_2_ (L × 10^3^)	+ 28.8	+ 1,036	+ 14.8–23.8	+ 533–856
CO_2_ (L × 10^3^)	+ 22.7	+ 816	+ 13.3–21.3	+ 479–768
H_prod_ (MJ)	+ 412.0	+ 14,832	+ 288.1–462.2	+ 10,371–16,640
Water (L)	+ 60.5	+ 2,180	+ 72.7–123.1	+ 2,619–4,432

### Oxygen consumption

**Figure 2:**
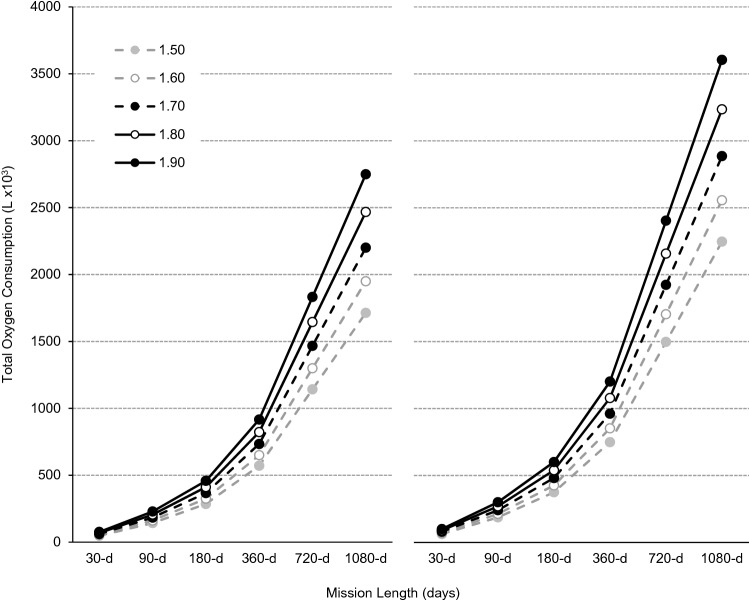
Total oxygen consumption (× 10^3^-L) without (left panel) and with (right panel) countermeasure exercise during exploration missions of 30-, 90-, 180-, 360-, 720- and 1,080-d for a four-person crew based on theoretical male astronaut populations with statures of 1.50-m (broken grey line, filled circles), 1.60-m (solid grey line, open circles), 1.70-m (broken black line, filled circles), 1.80-m (solid black line, open circles) and 1.90-m (solid black line, filled circles).

For a four-person all-male crew without, and with, the use of CM exercise, body size increased total O_2_ consumed from 47.6 × 10^3^-L for a ‘small-sized’ (1.50-m) crew to 76.4 × 10^3^-L for a ‘large-sized’ (1.90-m) crew during a 30-d mission, and from 1714 × 10^3^-L to 2,749 × 10^3^-L during a 1,080-d mission (Fig. 2). For a 30-d mission, CM exercise alone increased O_2_ consumed by 14.8 × 10^3^-L for a small-sized crew and by 23.8 × 10^3^-MJ for a large-sized crew, and by 533 × 10^3^-L and 856-L for a 1,080-d mission. Compared with a small-sized crew performing no CM exercise, a large-sized crew performing ISS-like CM exercise require an additional 52.5 × 10^3^-L of O_2_ per month (Table 4).

### Carbon dioxide production

**Figure 3:**
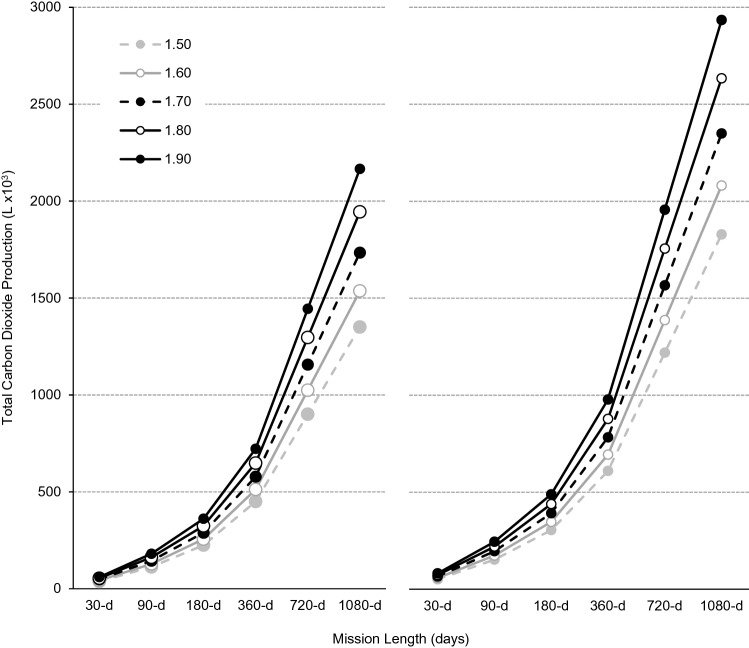
Total carbon dioxide production (× 10^3^-L) without (left panel) and with (right panel) countermeasure exercise during exploration missions of 30-, 90-, 180-, 360-, 720- and 1,080-d for a four-person crew based on theoretical male astronaut populations with statures of 1.50-m (broken grey line, filled circles), 1.60-m (solid grey line, open circles), 1.70-m (broken black line, filled circles), 1.80-m (solid black line, open circles) and 1.90-m (solid black line, filled circles).

For a four-person all-male crew without, and with, the use of CM exercise, body size increased total CO_2_ production from 37.5 × 10^3^-L for a ‘small-sized’ (1.50-m) crew to 60.2 × 10^3^-L for a ‘large-sized’ (1.90-m) crew during a 30-d mission, and from 1,350 × 10^3^-L to 2,166 × 10^3^-L during a 1,080-d mission (Fig. 3). For a 30-d mission, CM exercise alone increased CO_2_ production by 13.3 × 10^3^-L for a small-sized crew and by 21.3 × 10^3^-L for a large-sized crew, and by 479 × 10^3^-L and 768 × 10^3^-L for a 1,080-d mission. Compared with a small-sized crew performing no CM exercise, a large-sized crew performing ISS-like CM exercise produce an additional 44.0 × 10^3^-L of CO_2_ per month (Table 4).

### Heat production

**Figure 4:**
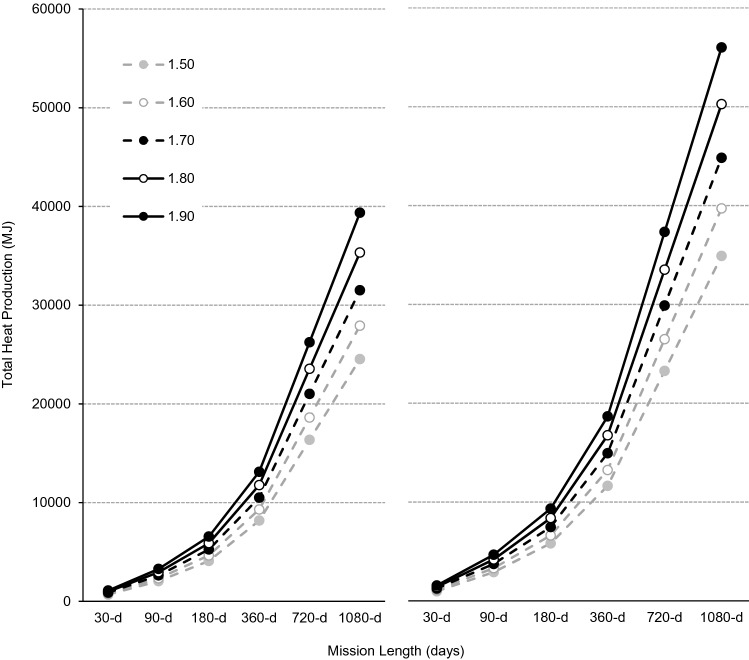
Total metabolic heat production (MJ) without (left panel) and with (right panel) countermeasure exercise during exploration missions of 30-, 90-, 180-, 360-, 720- and 1,080-d for a four-person crew based on theoretical male astronaut populations with statures of 1.50-m (broken grey line, filled circles), 1.60-m (solid grey line, open circles), 1.70-m (broken black line, filled circles), 1.80-m (solid black line, open circles) and 1.90-m (solid black line, filled circles).

For a four-person all-male crew without, and with, the performance of CM exercise, body size increased total H_prod_ from 681.6-MJ for a ‘small-sized’ crew (1.50-m) to 1,093.6-MJ for a ‘large-sized’ (1.90-m) crew during a 30-d mission, and from 24,539- to 39,371-MJ during a 1,080-d mission (Fig. 4). For a 30-d mission, CM exercise alone increased total H_prod_ by 288.1-MJ for a small-sized crew and by 462.2-MJ for a large-sized crew, and by 10,371-MJ and 16,640-MJ for a 1,080-d mission. Compared with a small-sized crew performing no CM exercise, a large-sized crew performing ISS-like CM exercise produce an additional 874.2-MJ of heat per month (Table 4).

### Water requirements for hydration

**Figure 5:**
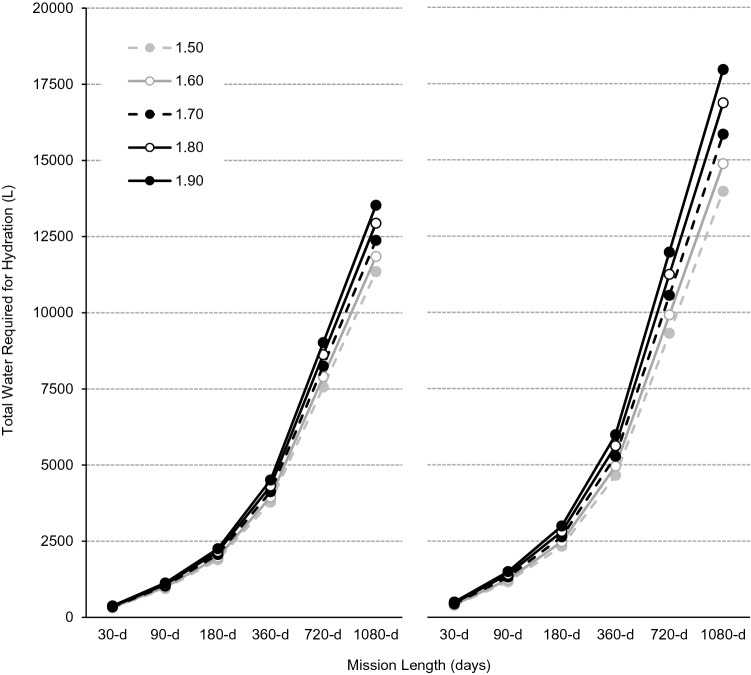
Total water requirements (L) without (left panel) and with (right panel) countermeasure exercise during exploration missions of 30-, 90-, 180-, 360-, 720- and 1,080-d for a four-person crew based on theoretical male astronaut populations with statures of 1.50-m (broken grey line, filled circles), 1.60-m (solid grey line, open circles), 1.70-m (broken black line, filled circles), 1.80-m (solid black line, open circles) and 1.90-m (solid black line, filled circles).

For a four-person all-male crew without, and with, the performance of CM exercise, body size increased total fluid needs from 315-L for a ‘small-sized’ (1.50-m) crew to 376-L for a ‘large-sized’ (1.90-m) large crew during a 30-d mission, and from 11,350- to 13,530-L during a 1,080-d mission (Fig. 5). For a 30-d mission, CM exercise alone increased water requirements by 72.7-L for a small-sized crew and to 123.1-L for a large-sized crew, and by 2,618-L and 4,432-L for a 1,080-d mission. Compared with a small-sized crew performing no CM exercise, a large-sized crew performing ISS-like CM exercise require an additional 183.6-L water per month for hydration (Table 4).

## Discussion

Based on our assumptions and methodological approach, the main findings of this study are that increasing theoretical male astronaut population height from 1.50- to 1.90-m increases resting 24-h TEE by + 44%, O_2_ consumed (+ 60%), CO_2_ (+ 60%) and metabolic heat (+ 60%) produced, and water required for hydration (+ 19%). Furthermore, performance of ISS-like CM exercise increases TEE (+ 29 to 32%), O_2_ consumed (+ 31%), CO_2_ (+ 35%) and metabolic heat (+ 42%) produced, and water requirements (+ 23 to 33%). For a four-person all-male crew, together, these differences translate in absolute terms to an additional 996-MJ energy, 52.5 × 10^3^-L of O_2_, 44.0 × 10^3^-L of CO_2_, 874-MJ of heat and 183.6-L of water per month.

In the early phase of ISS operations, crew received 1.8-kg/person/d of food, but mission guidelines state that the energy provided should be according on the estimated energy needs of the crewmember, based on body weight and height^19^. The estimations in this study suggest increasing theoretical male astronaut population body size alone increases the 24-h TEE requirement by 4.0-MJ/d (956-kcal). Based on the nutritional information from commercially-available, thermostabilized ‘ready-to-eat’ food analogous to current space food (Brown rice with chicken and vegetables: weight 250-g, energy: 365-kcal [1.53-MJ]; energy density: 1.46-kcal/g; volume: 340-cm^3^)^20^, the additional 55,742-MJ required for a 1,080-d mission with four male crew translates to 2,795-kg of food. Although a number of science experiments have grown food in space^21^, they are currently limited to small amounts of low-calorie salad crops. Thus, until a sufficient volume of high caloric-density food can be produced in space, the only option for future exploration missions is to launch food with the crew, potentially supplemented by additional supply missions, with significant implications for launch mass and mission architectures, and, when flown with the crew, on-board storage. For instance, based on the volume of a single 1.53-MJ portion of thermostabilized ‘ready-to-eat’ food^20^, the additional food required for a 1,080-d mission would occupy a volume of 3.8-m^3^.

Food and its packaging, in addition to human waste, are predicted to be greatest contributors to exploration mission waste^22,23^. Ewert and Broyan^22^ estimated that, for a 1-year mission (1,460-human days), a crew of four would need approximately 2,250-kg of food within 400-kg of packaging, with human waste estimated at 400-kg requiring a further 0.7-m^3^ of storage. Within the Gemini (space programme) food system, bite-size cubes of meat, fruit, dessert, and bread products were engineered to deliver 21.3-kJ/g (5.1-kcal/g), with the complete system offering approximately 2,890-kcal in 0.73-kg of packaged food^24^. However, the in-flight acceptability of these cubes quickly declined resulting in many returning uneaten^25^. Reductions in mass and volume are possible by increasing the proportion of energy from fat (currently up to 35% of total energy intake per NASA dietary guidelines), reducing water content and reducing reliance on energy ‘dilute’ foods such as beverages and vegetables. In ISS food, water contributes nearly 60% of total food weight, without any caloric value, resulting in thermostabilized pouches accounting for 65% of mass whilst providing only 30% of total calories^26^. Stoklosa^26^ reports that a 10% decrease in water and an increase in energy sourced from fat to 35%, yielded a mass saving of 22% (321-g/d). Substitution of standard menu items with one ‘meal replacement bar’ (400-kcal per 100-g bar) per crewmember, per day, saved 17% (240-g/d), whilst combining the two approaches saved 36% (529-g/d). However, both increasing fat and decreasing water content may negatively impact acceptability, while reducing reliance on energy dilute foods may also impact on the delivery of appropriate nutrients^26^.

Exercise is the cornerstone of the ISS CM programme and is likely to remain so on future exploration missions until an efficacious (across multiple physiological systems), safe and practical alternative is found^27^. As such, the provision of food for exploration missions will need to account for the higher energy expenditure to ensure energy balance is maintained, or body mass/fat losses are at least managed within acceptable limits. Although not measured directly, estimations in the present study suggest that current ISS CM exercise increases 24-h energy expenditure by between 2.6- and 4.1-MJ/d for theoretical male astronauts of 1.50-m and 1.90-m. Based on the nutritional information from the commercially-available food used above^20^, this would equate to a further 360- to 470-g of food per person, per day. Combined with the effect of increasing body size, CM exercise could require an additional 4,598- to 5,688-kg of food, occupying 6.2–7.7-m^3^, for a four-person all-male crew during a 1,080-d mission.

The current ISS CM exercise programme of 12 sessions/week of aerobic and resistance exercise, results in approximately 75-min (30-min aerobic, 45-min resistance) of exercise per day. However, High Intensity Interval Training (HIIT) or Sprint Interval Training (SIT), which involve much shorter (although higher intensity) periods of activity than traditional aerobic exercise, may offer alternative strategies to effectively stimulate the cardiorespiratory system^28^ and, by significantly reducing the total duration of exercise, could also reduce total energy expenditure. Despite higher intensities, HIIT and SIT protocols result in 100–250-kcal less energy expenditure compared with 40-min of cycling at 60–65% VO_2max_^29^. Further energy expenditure savings might also be made by reducing the number of intervals/sprints^28^. ISS resistance exercise is based on the traditional approach of ‘multiple sets of multiple repetitions’^16^, but recent evidence suggests that, while multiple sets may be optimal, significant benefits may be achieved from only a single set^30^. Alternative forms of exercise might also be effective in reducing energy expenditure. For instance, just 3- to 4-min of high-intensity jumping (comprising of approximately 50 jumps) six times per week using a supine pressure-cylinder-based sled was evaluated as a CM exercise during a long-term bed rest study^31^. This CM reportedly prevented bed rest-induced reductions in tibial bone mineral density and content, and muscle strength^32^, suggesting that it could be an effective alternative to resistance exercise. Moreover, jumping also prevented the bed rest-induced decrease in VO_2max_^32^ and results in comparable acute responses to running/cycling HIIT^33^. Therefore, although the energy expenditure associated with the jumping protocol is yet to be quantified, short duration (less than 25-min per week), sled-based jumping could conceivably replace both aerobic *and* resistance exercise in space, resulting in marked reduction in exercise activity thermogenesis (EAT)-related energy expenditure, as well as oxygen consumption and CO_2_ production.

Given the incompressibility of water, transportation in space vehicles from the ground has obvious implications for launch mass and volume, and subsequently storage. Thus, for exploration missions, the greater the number of crew and the longer the mission, the greater the challenge, unless water can be 100% recycled or acquired from a planetary body. This study estimates that, for missions lasting 30- to 1,080-d, daily water requirements for hydration are increased by both body size (+ 19% independent of mission length) and CM exercise (30-d: + 23%; 1,080-d: + 33%). In the complete absence of in-flight water recycling and/or in-situ extraction/creation, together, increased body size (resulting from an increase in stature from 1.50- to 1.90-m) and ISS-like CM exercise would require an additional 183.6-kg (0.1836-m^3^) of water to be stored and launched for each month of a mission for a four-person all-male crew.

Without recycling, water represents over 90% of the required life-support consumables for space missions, with a similar proportion of wastewater being classified as moderately, or slightly contaminated^34^. However, in-flight water recycling and generation as a by-product of other processes (e.g. fuel cell electricity production) can significantly reduce the need for water provision from the ground. The current ISS closed-loop system has an efficacy of approximately 93%^1^, reducing the net mass of water launched from Earth to support six crewmembers by 6,800-kg/year ^35^. Additional (2,500-L/year) water is provided on ISS by a Sabitier process-based^36^ system integrated into the ISS system^2,37,38^.

The primary source of O_2_ on ISS is provided via the electrolysis of water yielding O_2_ and hydrogen (vented overboard). Additional O_2_ is provided by the CO_2_ removal system, although only around 50% of the O_2_ is recovered^39^. To provide a closed-loop air revitalization system for future exploration missions, 75 to 90% of O_2_ must be recovered from CO_2_^40^ and efforts are underway to develop technologies that meet this requirement^39^. As O_2_ recovery is key to reducing (or even eliminating) reliance on ground supply, but is currently substantially less than 100% efficient^41^, an increased rate of oxygen consumption resulting from increased body size and CM exercise would increase the rate at which a fixed quantity of generated oxygen is depleted and no longer able to support optimal crew cognitive function and subsequently respiration.

Atmospheric CO_2_ management is also a critical element of life-support. The ISS CO_2_ management system requires crew maintenance every 3–6 months using replacement parts from Earth^42^ and is thus incompatible with long-duration exploration missions, which require a closed-loop air revitalization system that recovers 75–90% of O_2_ from CO_2_^40^. Such technologies are in development^43^, but to what extent the maintenance schedule of such technologies depends on the total amount of CO_2_ generated/removed and, therefore, the importance of factors such as body size and CM exercise, is currently unknown. Although the number of crew during exploration missions will likely be fewer than on ISS (four *vs*. six), the smaller pressurized volume of exploration vehicles/habitats could potentially increase the volatility of CO_2_ levels depending upon metabolic CO_2_ production and/or CO_2_ removal efficiency. The ISS system generally maintains CO_2_ partial pressures below 3-mmHg (depending on crew size/activity). Nevertheless, crew frequently report CO_2_-related symptoms such as headache^11^ and chronic CO_2_ elevation has been implicated in the spaceflight-associated neuro-ocular syndrome^44^.

Both the space vehicle/habitat, and its systems/payloads and the crew within, produce ‘waste’ heat, which must be circulated and removed to maintain thermal comfort, which is critical for human performance and wellbeing. In a habitat the size and complexity of ISS (338-m^3^)^4^, the relative contribution to internal heat load of the crew (even including CM exercise) compared with the station systems is minor. However, this contribution may increase (relatively) as vehicle size decreases. Lefeng et al.^45^ modelled that, in a 100-m^3^ volume, increasing crew metabolic activity from 80-W during sleep up to 240-W during moderate activity may have a marked effect on temperature. Thus, crew adhering to an intensive CM exercise programme in substantially smaller vehicles/habitats may result in a significant contribution of metabolic heat to the total internal heat load. ISS exercise sessions are typically brief (30–45-min), although some crew prefer to perform their two daily exercise sessions consecutively. As such, a ‘worse case’ scenario, would be all four exploration mission crew performing their twice daily exercise sessions consecutively, equivalent to a single person performing CM exercise for in excess of four hours. The present study estimates that, with a ‘large-sized’ (1.90-m) theoretical male astronaut crewmember, metabolic heat production by the total crew when CM exercise was being performed would be 1,388-J/s ([1 × 1,070-J/s during exercise] + [3 × 106-J/s at rest]). As with CO_2_ removal, to what extent the longevity (and thus maintenance schedules of key parts such as pumps) of exploration cooling systems will be affected by the total amount of heat, and hence the significance of body size and CM exercise effects, is currently unknown.

In this study, estimated sweat rates during exercise ranged from 10.1- to 17.1-mL/min. Sweat rates in excess of 8-mL/min have been considered undesirable in space because of the potential formation of a sheeting layer over the skin^46^. However, the maximum evaporative capacity of the environment modelled in this paper is twice that considered previously (for the Space Shuttle: 27 °C, 21 Torr [80 F/70% relative humidity])^46^, resulting in lower skin wettedness estimates and risk of sheeting. However, smaller habitable volumes and/or lower maximal evaporative potentials may increase humidity. Vehicular environmental control challenges may also be partially offset by personal cooling technologies. A variety of microclimate cooling garments have been used to reduce body heat storage during work performed while wearing protective clothing^47,48^. Modern conventional liquid cooling and ventilation garments worn for extra-vehicular activities (space walks) can remove between 100- and 200-W of metabolic heat, which is 20–30% of what has been modelled during exercise in this study. The use of a personal cooling garment with 100- to 200-W of cooling power during exercise would reduce body heat storage by increasing dry (sensible) heat exchange. As a result, a sweat rate of 15-g/min could be reduced to between 6- and 10-g/min. This would reduce the ‘worse case’ scenario for heat load management without the need for a change in the maximal evaporative capacity of the vehicle environment or reduction in exercise intensity.

The most obvious limitation of this study is the necessity to make a number of assumptions about the components of TEE in microgravity as a result of the absence of spaceflight data, although in-flight studies are underway^49^. The typical thermic effect of food (TEM) profile, for example, may be delayed due to a reduction in gastrointestinal transit rate in microgravity^50^, although this is unlikely to have a significant impact on TEE. Non-exercise activity thermogenesis (NEAT) was assumed to be minimal, but not negligible, resulting in a Physical Activity Level (PAL) estimate of 1.4. Exactly how physically active (excluding CM exercise) crew will be during future exploration missions is unknown, and likely depends of the size of the vehicle/habitat, the demands of operating it and any other operational activity requirements. As a result, a PAL of 1.4 might be an under- or overestimate of NEAT. Surface operations will, of course, increase NEAT, but to what extent is unknown, but will depend on a range of factors such as the activity, protective environment (i.e. habitat or space suit), and the surface and gravitational environment^51^, hence surface operations were not included in the calculations.

Secondly, although it was assumed that the elevated atmospheric level of CO_2_ would have no effect on metabolism at rest or during exercise, an effect of a CO_2_ concentration of ~ 0.5% CO_2_ on the ventilatory response to exercise cannot be ruled out^52^. An additional limitation is the modelling of resistance exercise as a second bout of aerobic exercise in the absence of appropriate validated equations for calculation of energy expenditure and water requirements. In fact, single bout resistance exercise energy expenditure estimates range from 2.7- to 11-kcal/min^53^, presumably reflecting factors such as number of sets and repetitions, and types of exercise. Such variation will of course influence VO_2_, VCO_2_, water needs and heat production estimates. A fourth limitation is that the equation used to predict exercise sweat losses^54^ in this study, which also requires estimates of the biophysical properties of clothing^55^, was not explicitly designed with microgravity in mind. However, it is based upon biophysical principles that have been applied previously to estimate the exercise sweating response to exercise in space^46^ yielding sweating rates in this study consistent with those estimated using a pure heat balance approach^46^.

This paper focused only on male astronauts and, as a result, used available data and equations (e.g*.* VO_2max_, resting metabolic rate [RMR]) specific to males. However, although historically a high percentage of astronaut populations have been male, five of the 11 astronauts selected by NASA in 2019 were female. As such, given that there are physiological differences between males and females in terms of body composition and metabolism^17^, and responses to the spaceflight environment^18^, but the effects of these differences on resource requirements have not been evaluated, future studies should repeat these calculations with a specific focus (assumptions and equations) on females and compare the results of those with males across an identical height range. Finally, in order to provide an estimate of VO_2max_ (in L/min) for subsequent calculations, in the absence of the availability of individual astronaut data, it was necessary to make an assumption about relative (in mL/kg/min) VO_2max_ from mean values published from an astronaut population^56^. This assumes geometric similarity between the different body sizes, but such similarity is not evident for muscle mass in both athletic and non-athletic populations^57^, which has implications for metabolism, energy expenditure and VO_2max_^58^. As such, the assumption of geometric similarly in this paper may mask potential variability between individuals and is likely to result in an underestimate of metabolism and energy expenditure in the larger theoretical populations. With access to individual body masses and direct measures of VO_2max_ from a large group of astronauts of varying body sizes, future investigations should evaluate the use of multi-parameter allometric scaling techniques. This should also include separate analysis of male and female astronauts to account for the possible influence of differences in body somatotype/composition and metabolism^17^.

Using a stature range of 1.50- to 1.90-m and based on assumptions about the crew (all male; age: 40 years old; body mass index (BMI): 26.5-kg/m^2^; resting VO_2_ and VO_2max_: 3.3- and 43.4-mL/kg/min, and geometric similarity across the stature range) and their energy expenditure, several of which should be refined as, and when, appropriate spaceflight data become available, this theoretical study provides the first insight into the potential implications of body size and the performance of ISS-like CM exercise upon the provision of life-support required to sustain humans during exploration missions. Novel technological approaches are required and should be evaluated considering crew composition (body size) and predicted exercise CM regimes. Although such technologies will reduce the absolute magnitude of some effects of body size and CM exercise, relative differences will remain. Whilst closed-loop regenerative O_2_, water and CO_2_ management systems may be possible, consideration of strategies to minimize and meet crew metabolic energy needs (and dissipation), estimated in this study to increase with body size and CM exercise by 966-MJ per month and requiring an additional 5,688-kg of food occupying 7.7-m^3^ during a 1,080-d mission, is required.

## Methods

### Assumptions and rationales

For the purpose of all calculations in this paper, and to provide upper and lower limits for the examining the effects of body size, the following has been assumed.

#### Assumptions about the missions and vehicle/habitat

1. Mission durations will range from 30-d (transit-out, Lunar orbit, transit-back) up to 1,080-d (transit-out, prolonged Martian orbit, transit-back), all without human surface exploration (i.e. crew will remain inside vehicles/habitats for the entire mission);
2. Missions will be crewed by four male astronauts.
3. The environment inside the vehicle is comparable to that currently on ISS: 760-mmHg barometric pressure, 20.9% oxygen, ~ 0.5% CO_2_, balance nitrogen at 101.3-kPa (14.7-psi), mean temperature 22 °C, 55% relative humidity^10^.
4. The elevated CO_2_ concentration inside the space vehicle has no effect on metabolism, either at rest, or during exercise. The respiratory response to 1.0% CO_2_-induced acidosis is a 3.5-mL/min increase in minute ventilation and thus VO_2_^59^, although to our knowledge, the effect of 0.5% CO_2_ is unknown. However, given that resting minute ventilation tends to return to baseline values by the second to third week of exposure to 1.0% CO_2_^60^, it is assumed that there is no significant chronic ventilatory response to 0.5% CO_2_.
5. Airflow experienced by the crew member (provided by the vehicle/habitat ventilation system) during CM exercise is 0.5 m/s.

#### Assumptions about the crew and their physiology at rest

1. Crew stature ranges from 1.50- (“small”) to 1.90-m (“large”) (59.1–74.8″), which is representative of historical and current stature requirements (for both males and females) for NASA, and the European (ESA) and Canadian (CSA) Space Agencies^7–9^_._
2. Crew are geometrically similar across this stature range.
3. Independent of stature, all crew have, and maintain, a BMI of 26.5-kg/m^2^, calculated from the mean height (1.755-m) and body mass (81.6-kg) of a group of 30 male astronauts^56^;
4. Crew are all 40 years old. Neither NASA or CSA include age restrictions in their selection requirements, and ESA only state a ‘preferred age range’ of 27–37 years old^8^. NASA astronaut candidates selected in the past have ranged from 26 to 46 years old, with the current average being 34 years old^61^. As such, when accounting for the prolonged training required for an exploration mission, it is assumed that all crew will be at least 40 years old;
5. As no 24-h TEE, or its components (RMR, and non-resting energy expenditure, composed of NEAT, EAT and TEM) are currently available from spaceflight, the following are assumed:
  - RMR is equivalent to that on Earth, and does not change during the mission;
  - NEAT is minimal but not negligible, resulting in a PAL of 1.4 (equivalent to a very sedentary lifestyle on Earth) excluding CM exercise (see below for assumptions/calculations related to EAT for astronauts performing CM exercise). PAL estimates in space range from 1.2 (Vostok missions)^62^ to as much as 1.7–1.75 (Skylab missions)^63,64^. Values of 1.2 are associated with low levels of activity (in fact no formal exercise was prescribed during Vostok missions) and, therefore, presumably reflects microgravity-induced hypokinesis, whereas values of 1.7–1.75 reflect intensive performance of CM exercise by Skylab crew. Thus, whilst exploration vehicles/habitats have yet to be fully defined, they will likely possess sufficient internal volume to allow free movement with crew being required to perform light operational activities (e.g. scientific experiments) each day, resulting in a moderate energy cost. Therefore, whilst formal CM exercise is considered separately (see below), calculated RMR, and resting VO_2_ and CO_2_ values were multiplied by 1.4;
  - EAT is related to the use of CM exercise (see below);
  - TEM requires 206 kcal (0.87 MJ) of energy expenditure. 206-kcal is the average (multiplied by three to account for three meals per day) energy expenditure associated with the ingestion of four representative test meals (600-kcal and 1,200-kcal; high carbohydrate-low fat, and low carbohydrate-high fat)^65^.
6. VO_2_ at rest (RMR) is 3.3-mL/kg/min. Resting VO_2_ is generally accepted to be approximately 3.5-mL/kg/min^66–68^, however, Kozey et al.^69^ reported figures of 3.3- and 3.2-mL/kg/min in groups aged 33- and 45-years old. Kozey et al.^69^ also showed that resting VO_2_ is influenced by estimated (via a prediction equation based on age, height, sex, body mass, and physical activity) fitness level^70^, with resting VO_2_ being 2.7, 3.2, 3.3, 3.5 and 3.3-mL/kg/min per ascending quintile. Pre-flight astronaut aerobic fitness varies considerably between individuals, however, mean ± standard deviation values of 43.4 ± 7.8 and 40.5 ± 7.0 mL/kg/min have been reported for men and women, respectively^56^. Such values place male and female crew aged 30- to 50-years, within Kozey et al.’s^69^ fifth quintile (3.3-mL/kg/min);
7. Respiratory exchange ratio (RER) at rest is 0.788. Human RER ranges considerably at rest but, based on these data of McNeil et al.^71^ and Weyer et al.^72^, has an average of 0.788.
8. Resting VCO_2_ is 2.6-mL/kg/min. Resting VCO_2_ is commonly assumed to be 200-mL/min. However, resting VCO_2_ is dependent upon energy substrate utilization reflected in the RER (VCO_2_/VO_2_). Thus, assuming a resting VO_2_ of 3.3-mL/kg/min and a resting RER of 0.788, yields a resting VCO_2_ of 2.6-mL/kg/min.
9. Respiratory water losses are balanced by metabolic water production, and thus can be discounted, whereas transcutaneous water loss is only considered for total body water balance^73^.
10. Protein (95-g/d), sodium (4,320-mg/d) and potassium (3,062-mg/d) intake data are as reported in ISS Expeditions 26–37^74^.
11. Core temperature is 37 °C.

#### Assumptions about the crew and their physiology during exercise

Countermeasure exercise on ISS currently consists of two sessions per day (1 × 30–45-min of aerobic and 1 × 45-min of resistance), 6-d/week, with target workloads for steady-state and interval-type aerobic protocols of 75–80% and 60–90% VO_2max_^16^. However, due to the challenge of modelling non-steady state exercise^75,76^ such as intermittent, high intensity resistance exercise as used on ISS, for the purpose of this paper, CM exercise will be modelled as 30-min of steady-state aerobic exercise at 75% VO_2max_, *twice* per day, 6-d/week. The assumptions used are as follows:

12. Crew have a relative VO_2max_ of 43.4-mL/kg/min, as reported for a group of 30 male astronauts^56^;
13. During aerobic exercise crew wear light sports clothing (shorts, t-shirt and socks), with thermal and evaporative resistances equal to 0.06 m^2^ × °C/W and 0.01 m^2^ × kPa/W, respectively^55^.
14. VO_2_ during exercise is 32.6-mL/kg/min (i.e. 75% of 43.4-mL/kg/min). For the purpose of calculation simplification, any warm-up or cool-down periods have been excluded;
15. RER during exercise at 75% VO_2max_ is 0.898. In the study of Scott et al.^77^, subjects exercising at 75 ± 3% VO_2max_ in a fasted state had a mean RER of 0.898. Pre-exercise food consumption may alter substrate utilization, and thus RER during exercise. However, Bergman and Brooks^78^ showed that, whilst food intake significantly increased RER at exercise intensities up to 59% VO_2peak_, RER was unaffected at 75% VO_2peak_.
16. Based on a VO_2_ of 32.6-mL/kg/min and an RER of 0.898, VCO_2_ during exercise is 29.2-mL/kg/min.
17. Excess post-exercise oxygen consumption, energy expenditure and VCO_2_ will be 6% of that consumed during exercise itself. No data (% of energy expended during exercise) for 30-min at 75% VO_2max_ is available, but Bahr et al.^79^ report figures of 5.1% and 6.8% for 20- and 40-min of exercise at 70% VO_2max_ and the mean of those two values has been used;
18. RER is equivalent to that at rest during recovery from exercise. Immediately following exercise at 70% VO_2max_, there is a brief increase in RER followed by a rapid decline, lasting around 10-min in total^80^. Initially, the decline in VO_2_ exceeds the rate of ventilation reduction (resulting in more CO_2_ release from the lungs), after which ventilation rates falls in line with VO_2_ albeit limited by the drive to restore acid–base balance thereby promoting CO_2_ retention. As such, RER during this brief period may not be an accurate index of energy substrate utilization, after which, RER is at, or very close to, that measured pre-exercise^80^.

## Calculations

1. RMR was calculated using the Revised Harris–Benedict equation^81^ for males, where:
  $$\begin{aligned} RMR \, & = \, 88.362 \, + \, \left( {13.397 \, \times \, weight \, in \, kg} \right) \, + \, \left( {4.799 \, \times \, height \, in \, cm} \right) \\ & \quad - \, \left( {5.677 \, \times \, age \, in \, years} \right). \\ \end{aligned}$$
2. BMI^82^ was calculated as:
  $$BMI\,\left( {{\text{kg}}{/}{\text{m}} } \right) \, = \, body\,mass\,\left( {{\text{kg}}} \right) \, \cdot \, height\,\left( {\text{m}} \right)^{ - 2}$$
3. Energy expenditure (EE) during exercise (kcal/min) was estimated using the Weir equation^83^, where:
  $$EE \, = \, 3.94 \, VO_{2} ({\text{mL}}{/}\text{min} ) + \, 1.11 \, VCO_{2} ( {{\text{mL}}{/}\text{min} })$$
4. Body surface area (BSA) was calculated using the formula of Du bois and Du bois^84^:
  $$BSA \, = \, 0.007184 \, \cdot \, height \, \left( {in \, metres} \right)^{0.725} \cdot \, weight \, \left( {in \, kg} \right)^{0.425}$$
5. Insensible water needs (IWN), dietary solute load (DSL) and urine volume to maintain a 24-h urine volume at a concentration of 600-mmol/kg (UV_600_)^85^ was calculated as:
  $$\begin{aligned} IWN \, & = \, \left( {0.4 \, \cdot \, Energy \, Needs \, \left[ {in\,{\text{kcal}}} \right]} \right){/}1000 \\ DSL \, & = \, \left( {Protein \, intake \, \left[ {{\text{g}}{/}{\text{d}}} \right]{/}0.175} \right) \, + \, \left( {2* \, \left( {Na \, intake \, \left[ {{\text{mg}}{/}{\text{d}} } \right]{/}23 \, + \, K\,intake \, \left[ {{\text{mg}}{/}{\text{d}} } \right]{/}39} \right)} \right) \\ UV_{600} & = \, DSL{/}600 \\ \end{aligned}$$
6. The rate of metabolic heat production (M_prod_) at rest was calculated as:
  $$M_{prod} \left( {{\text{J}}{/}{\text{s}}} \right) \, = \, \left( {VO_{2} \left[ {{\text{mL}}{/}{\min}} \right] \, \times \, Thermal\,Equivalent\,of\,O_{2} \,at\,RER} \right){/}0.01433$$
  where the thermal equivalent of O_2_ at resting RER (0.788) is 4.788-kcal/min/L.
7. M_prod_ during exercise was calculated using the above formula, where the thermal equivalent of O_2_ at exercising RER (0.898) is 4.924-kcal/min/L.
8. Using an M_prod_ adjusted for the rate of external work during cycling (M_prod_ − *Rate of External Work *[*W*]), where W was assumed to be 20% of M_prod_ for non-cyclists^86^, sweat rate during exercise was calculated by first, using partitional calorimetry formulae for body heat balance to derive the requirement for evaporative cooling (E_req_) and the maximal evaporative capacity of the environment (E_max_)^87^. The equation of Gonzalez et al.^54^ was then applied to estimate steady-state exercise sweating rate. No allowance was made for the thermal inertial lag in sweating onset as it is generally balanced by the reciprocal thermal decay post-exercise^88^.
